# Non-traumatic coma in young children in Benin: are viral and bacterial infections gaining ground on cerebral malaria?

**DOI:** 10.1186/s40249-022-00956-2

**Published:** 2022-03-14

**Authors:** Josselin Brisset, Karl Angendu Baki, Laurence Watier, Elisée Kinkpé, Justine Bailly, Linda Ayédadjou, Maroufou Jules Alao, Ida Dossou-Dagba, Gwladys I. Bertin, Michel Cot, Farid Boumédiène, Daniel Ajzenberg, Agnès Aubouy, Sandrine Houzé, Jean-François Faucher, NeuroCM Group, Dissou Affolabi, Nicolas Argy, Bibiane Biokou, Jean-Eudes Degbelo, Calavi, Benin, Philippe Deloron, Latifou Dramane, Jérémy Fraering, Emilie Guillochon, Sayeh Jafari-Guemouri, Ludivine Houzé, Valentin Joste, Claire Kamaliddin, Anaïs Labrunie, Yélé Ladipo, Thomas Lathiere, Achille Massougbodji, Audrey Mowendabeka, Jade Papin, Bernard Pipy, Pierre-Marie Preux, Marie Raymondeau, Jade Royo, Darius Sossou, Brigitte Techer, Bertin Vianou

**Affiliations:** 1 Infectious Diseases and Tropical Medicine Department, Limoges University Hospital, 2 Avenue Martin Luther King, 87000 Limoges, France; 2 Inserm UMR 1094, IRD U270, EpiMaCT - Epidemiology of chronic diseases in tropical zone, Institute of Epidemiology and Tropical Neurology, OmegaHealth, Univ. Limoges, CHU Limoges, Limoges, France; 3 Center for Research in Epidemiology and Population Health (CESP), INSERM U1018, Paris-Saclay University, UVSQ, Montigny-Le-Bretonneux, France; 4 Epidemiology and Modeling of Bacterial Evasion to Antibacterials Unit (EMEA), Institut Pasteur, 25-28, Rue du Dr. Roux, 75724 Paris Cedex 15, France; 5 Paediatric Department, Calavi Hospital, Calavi, Benin; 6 UMR261 MERIT, IRD, Université de Paris, 75006 Paris, France; 7 French Malaria Reference Center, Hôpital Bichat, APHP, Paris, France; 8 Paediatric Department, Mother and Child University and Hospital Center (CHU-MEL), Cotonou, Benin; 9 Clinical Research Institute of Benin (IRCB), Abomey Calavi, Benin; 10 UMR152 PHARMADEV, Université de Toulouse, IRD, UPS, Toulouse, France; 11 Parasitology Laboratory, Hopital Bichat-Claude-Bernard, APHP, Paris, France

**Keywords:** Non-traumatic coma, Cerebral malaria, Co-infection, Central nervous system infection, West Africa

## Abstract

**Background:**

While malaria morbidity and mortality have declined since 2000, viral central nervous system infections appear to be an important, underestimated cause of coma in malaria-endemic Eastern Africa. We aimed to describe the etiology of non-traumatic comas in young children in Benin, as well as their management and early outcomes, and to identify factors associated with death.

**Methods:**

From March to November 2018, we enrolled all HIV-negative children aged between 2 and 6 years, with a Blantyre Coma Score ≤ 2, in this prospective observational study. Children were screened for malaria severity signs and assessed using a systematic diagnostic protocol, including blood cultures, malaria diagnostics, and cerebrospinal fluid analysis using multiplex PCR. To determine factors associated with death, univariate and multivariate analyses were performed.

**Results:**

From 3244 admissions, 84 children were included: malaria was diagnosed in 78, eight of whom had a viral or bacterial co-infection. Six children had a non-malarial infection or no identified cause. The mortality rate was 29.8% (25/84), with 20 children dying in the first 24 h. Co-infected children appeared to have a poorer prognosis. Of the 76 children who consulted a healthcare professional before admission, only 5 were prescribed adequate antimalarial oral therapy. Predictors of early death were jaundice or increased bilirubin [odd ratio (*OR*)= 8.6; 95% confidential interval (*CI*): 2.03–36.1] and lactate > 5 mmol/L (*OR* = 5.1; 95% *CI*: 1.49–17.30). Antibiotic use before admission (*OR* = 0.1; 95% *CI*: 0.02–0.85) and vaccination against yellow fever (*OR* = 0.2, 95% *CI*: 0.05–0.79) protected against mortality.

**Conclusions:**

Infections were found in all children who died, and cerebral malaria was by far the most common cause of non-traumatic coma. Missed opportunities to receive early effective antimalarial treatment were common. Other central nervous system infections must be considered in their management. Some factors that proved to be protective against early death were unexpected.

**Supplementary Information:**

The online version contains supplementary material available at 10.1186/s40249-022-00956-2.

## Background

Neurological involvement in severe malaria is revealed by convulsions and/or coma [1]. These clinical features do not help with distinguishing cerebral malaria (CM) from other severe brain injuries, such as viral and bacterial central nervous system (CNS) infections [2, 3], or other encephalopathies [4]. Mixed infections also occur and autopsy studies [5] have revealed cases of lethal meningitis in children with *Plasmodium falciparum* parasitemia. Both CM and CNS infections are life-threatening conditions that require the administration of adequate medications [1, 2, 6]. In resource-limited countries, however, investigating the etiologies of non-traumatic coma is challenging. Therefore, in areas of high malaria transmission, non-traumatic comas are commonly managed as CM cases [3]. A few years after the launch of the Roll Back Malaria program, a prospective cohort study in Malawi showed that viral CNS infection is an important cause of hospital admission and death in children [7].

Implementation in 2000 of the World Health Organization Roll Back Malaria program led to a 36% decline in the worldwide annual incidence of malaria and a 60% decline in the annual death rate [8]. Since 2015, however, progress has stalled. Furthermore, these epidemiological trends are heterogeneous, with some countries reporting dramatic improvements, and others not. Forty countries had a higher age-standardized incidence in 2019 than in 2015 [9]. Although malaria eradication within a generation is the goal [10], the number of deaths related to malaria may remain high, especially in areas of West and Central Africa.

Our present work is part of a larger project called NeuroCM [11, 12], whose main goal is to identify the causative and remedial factors of neuroinflammation in the context of CM. From the data collected in this prospective study, we undertook a retrospective analysis of the determinants of death in small children admitted with non-traumatic coma in South Benin, focused on the etiologies, clinical history, clinical and biological characteristics at admission, and management of the children.

## Methods

### Study design and participants

This prospective study was conducted at two university hospitals: University Hospital of Abomey Calavi/Sô-Ava (CHUZ-AS) and University Hospital of Mother and Child, Lagune (CHU-MEL) in Cotonou, Benin (Additional file 1: Fig. S1). A clinical research physician in each hospital was specifically trained on the protocol and dedicated to NEUROCM study management.

From March 1 to November 30, 2018, children aged between 24 and 71 months with non-traumatic coma and a negative human immunodeficiency virus (HIV) rapid diagnostic test (RDT) result were enrolled. Coma was defined based on a Blantyre Coma Score (BCS) of ≤ 2 [13].

The exclusion criteria were the absence of parental consent, pre-existent neurological disease, and traumatic or toxic coma.

### Ethics review and approval

Ethics approval for the NeuroCM study was obtained from *National Ethics Committee for Health Research of Benin* (n°67/MS/DC/SGM/DRFMT/CNERS/SA; 10/17/2017). The NeuroCM study was approved by Ethics Advisory Committee of Institut de Recherche pour le Développement (IRD; 10/24/2017).

Written informed consent was obtained from the parents or guardians of all included children.

### Diagnostic assignment

CM was defined as coma, with *P. falciparum* infection evidence on blood smear and/or PCR, and no other known cause of coma (e.g., acute bacterial meningitis, coma related to hypoglycemia reversed by glucose infusion, status epilepticus) [14]. Non-malarial non-traumatic coma was defined as coma and *Plasmodium* infection detected neither in thick blood smear nor PCR. Coinfection was defined as coma with *P. falciparum* on blood smear or PCR and evidence of another infectious pathogen.

Medical charts including clinical data and medical history, drug intake declared before admission, and biological data with microbiology (including those from tests performed retrospectively) were reviewed by an independent panel of three experts (two clinicians and a biostatistician) to assign a final diagnosis.

### Management

All children had their medical history recorded and underwent a physical examination at enrollment. The following data were collected using an online standardized form: demographic characteristics, vaccination status, healthcare pathway, clinical course and treatments, and clinical features and outcome. On enrollment, patients underwent an initial blood draw for analysis including a blood culture.

A lumbar puncture was performed as soon as possible if the patient was sufficiently clinically stable. All children with CM received intravenous artesunate treatment. Clinicians responsible for the children had access to other emergency treatments (ceftriaxone, anticonvulsants, glucose, fluids, blood transfusions), prescription of which was at the physician’s discretion.

The vital status at the end of hospitalization was used in the analyses to sort the patients into two groups: those who improved and were discharged from the hospital (“Survived”) vs those who died during hospitalization (“Died”).

### Laboratory testing

Thick and thin blood smear analysis, complete blood counts, and biochemistry analysis were performed on site. Parasitemia was confirmed at Clinical Research Institute of Benin (IRCB). Blood culture, Gram staining, and bacterial culture of cerebrospinal fluid were performed in a university hospital reference laboratory (National Hospital and University Center of Cotonou).

Additional laboratory tests were performed retrospectively on blood samples and cerebrospinal fluid samples collected at admission:

- Cerebrospinal fluid specimens were processed for adenovirus PCR (RealStar® Adenovirus PCR kit 1.0, Altona Diagnostics, France) and for *Escherichia coli*, *Hemophilus influenzae*, *Listeria monocytogenes*, *Neisseria meningitidis*, *Streptococcus agalactiae*, *S. pneumoniae*, cytomegalovirus, enterovirus, herpes simplex virus (HSV) 1, HSV2, human herpesvirus 6, parechovirus, varicella zona virus, *Cryptococcus neoformans*/*C. gattii* with the FilmArray® Meningitis/Encephalitis Panel (BioMérieux, France).
- PCR for the detection of dengue virus, Chikungunya virus, West Nile virus, *Plasmodium* spp., *Rickettsia* spp., *Leptospira* spp., *Salmonella* spp. (FTD Tropical Fever core PCR, Fast Track Diagnostics, Luxembourg) and *P. falciparum* (FTD Malaria differentiation, Fast Track Diagnostics, Luxembourg) and IgM measles (Measles IgM Capture EIA, Clin-Tech Limited, UK) were performed on the blood samples.

### Statistical analysis

For descriptive analysis, quantitative variables are presented as the means ± standard deviations or medians ± interquartile ranges, and qualitative variables are shown as frequencies (percentages).

Comparisons between groups for qualitative variables were conducted using Pearson’s *χ*^2^ test or Fisher’s exact test.

Intergroup comparisons of quantitative variables were conducted using Student’s *t*-test when applicable and the Mann–Whitney U test when normality assumption was not met.

Logistic regression analyses were performed to identify factors associated with mortality during hospital care. Variables with a *P*-value < 0.20 in univariate analysis were included in the multivariable logistic regression model, except for the center variable, which was included whatever its *P*-value. The final model was determined using a manual backward-selection procedure. When appropriate, interactions between variables were tested. A two-tailed *P* < 0.05 in the multivariable model was taken to denote significance.

All analyses were conducted using SAS software version 9.4 (SAS Institute Inc., Cary, NC).

## Results

### Study population

**Fig. 1:**
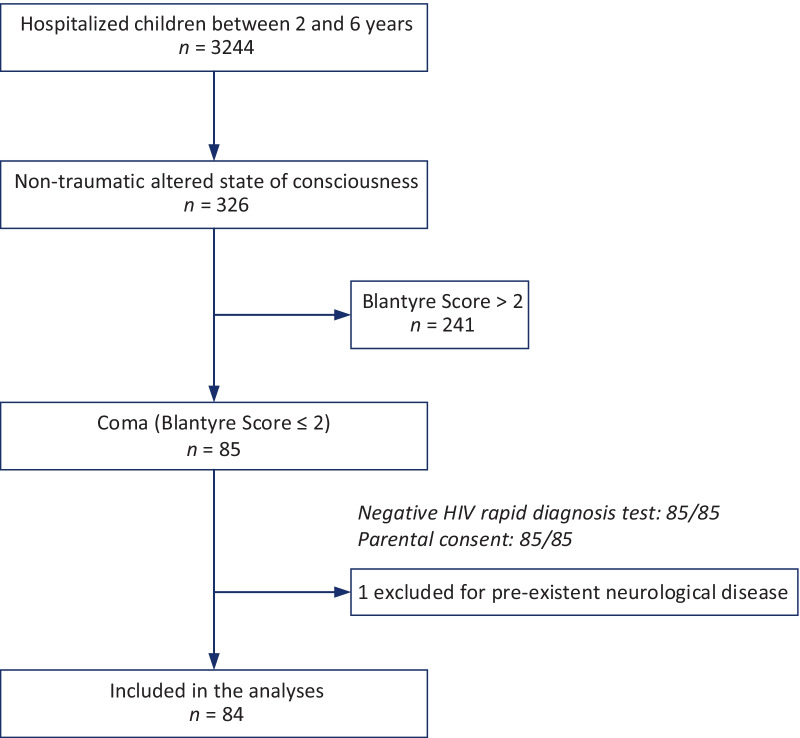
Flow diagram of the study participants

From March 1 to November 30, 2018, 326 children between 2 and 6 years of age were admitted to hospital with a non-traumatic altered state of consciousness, of whom 85 had a BCS of ≤ 2 (Fig. 1). All 85 patients had a negative HIV RDT result and were enrolled with parental or guardian consent. One patient was excluded for protocol violation because he suffered from a pre-existent neurological disease that was not declared by the guardian at admission, leaving 84 children (48 in CHUZ-AS, 36 in CHU-MEL). Fifty of them were females (male-to-female ratio: 0.68), with a mean age of 43 ± 13 months.

### Vaccination

Vaccination rates were high (> 80%) in both groups for the Bacille Calmette-Guérin (BCG; tuberculosis) vaccine; oral polio vaccine; diphtheria, tetanus, and pertussis vaccine; hepatitis B vaccine; *H*. *influenzae* type b vaccine; and pneumococcal conjugate vaccine (Table 1). The rates of measles, mumps and rubella, and yellow fever vaccination were lower in the children who died compared to the children who survived (62.5% vs 81.4% and 54.2% vs 77.2% respectively, Table 1).

**Table 1:** Vaccination and care pathway

	Survived (*n* = 59^a^)	Died (*n* = 25^a^)		*P*-value
*Vaccination*				
BCG	57/58 (98.3)	24/24 (100)		1.00
OPV	56/58 (96.6)	23/24 (95.8)		1.00
DTP-HepB-Hib	51/57 (89.5)	23/24 (95.8)		0.67
PCV13	50/57 (87.7)	20/24 (83.3)		0.72
MMR	48/57 (84.2)	15/24 (62.5)		0.10
YF	44/57 (77.2)	13/24 (54.2)		0.06
*Healthcare before hospital admission*^*b*^				
Heath center	51 (87.9)	23 (92.0)		0.72
Biomedicine consultation	54 (91.5)	22 (88.0)		0.69
Traditional healer consultation	23 (39.0)	11 (44.0)		0.67
Hospitalization	23 (39.0)	6/24 (25.0)		0.23
*Treatments before hospital admission*^*b*^				
Antibiotics	18 (30.5)	2 (8.0)		**0.03**
Anti-malarial drugs	22 (37.2)	8 (32.0)		0.64
Anti-epileptic drugs	4 (6.8)	0 (0.0)		0.31
Blood transfusions	2 (3.4)	0 (0.0)		1.00
*Means of transport*				
Car	9 (15.3)	4 (16.0)		1.00
Motorcycle	50 (84.8)	21 (84.0)		1.00
Travel time, hours, mean (*SD*)	1.05 (0.71)	1.10 (0.57)		0.39
Duration of symptoms before admission, days, mean (SD)	4.68 (2.24)	4.72 (2.46)		0.85

Data are numbers (%) of children, unless otherwise indicated

*SD* standard deviation, *BCG* Bacille Calmette-Guérin (tuberculosis) vaccine, *OPV* oral polio vaccine, *DTP* diphtheria, tetanus, and pertussis vaccine, *HepB* Hepatitis B vaccine, *Hib* Hemophilus influenzae type b vaccine, *PCV13* Pneumococcal conjugate vaccine (13-valent), *MMR* measles, mumps, and rubella vaccine, *YF* yellow fever vaccine

^a^For variables for which the number of children examined is less than the total number listed for the group, the numbers for that variable and group examined are noted in the table

^b^Between disease onset and admission

### Healthcare pathway

#### Travel time and means of transport

The majority of the children (84.5%) traveled from home by motorcycle. There was no significant difference in travel time—around 1 h on average—between the two groups (Table 1).

#### Duration of symptoms before admission

Fever was almost always present (98.8%) before admission, and in those with fever, it had been progressing for an average of 4.3 (± 1.8) days. The average duration of coma before admission to the hospital was 1.2 (± 0.5) days. The duration of symptom progression before admission was similar in the two groups (Table 1).

#### Medical care

Before hospital admission, 90.5% (76/84) of the children were seen by a healthcare provider (45/84 one visit, 27/84 two visits, 4/84 three or more visits), often in a health center (Table 1). Also, 40.5% (34/84) consulted traditional healers (16/84 one visit, 5/84 two visits, 13/84 three or more visits). Twenty-nine (34.5%) had been hospitalized prior to returning with impaired consciousness.

### Characteristics at admission

Data comparing the clinical and biological signs of severity at admission in the two groups are presented in Table 2. A BCS ≤ 1 was associated with higher mortality in a univariate analysis.

**Table 2:** Clinical manifestations and laboratory indices of severity at admission

	Survived (*n* = 59)		Died (*n* = 25)		*P*-value
Blantyre Coma Score					**0.004**
0 or 1	14 (23.7)		14 (56.0)		
2	45 (76.3)		11 (44.0)		
Prostration^a^	39 (66.1)		17 (68.0)		0.87
Multiple convulsions^b^	29 (49.2)		11 (44.0)		0.62
Respiratory distress or acidotic breathing	11 (18.6)		10 (40.0)		**0.04**
Shock, systolic blood pressure < 50 mmHg	2 (3.4)		2 (8.0)		0.58
Jaundice or increased bilirubin > 50 μmol/L	18 (30.5)		17 (68.0)		**0.002**
Abnormal bleeding^c^	1 (1.7)		2 (8.0)		0.21
Hypoglycemia (glucose < 2.2 mmol/L)	13 (22.0)		15 (60.0)		**0.0007**
Acidosis (plasma bicarbonate < 15 mmol/L)	20 (34.5)		20 (80.0)		**0.0001**
Hyperlactatemia (venous plasma lactate > 5 mmol/L)	27 (46.5)		21 (84.0)		**0.002**
Severe anemia (hemoglobin < 50 g/L, hematocrit < 15%)	22 (37.3)		10 (40.0)		0.81
Renal impairment (serum creatinine > 265 μmol/L	0 (0.0)		2 (9.1)		0.07

Data are numbers (%) of children, unless otherwise indicated

^a^Generalized weakness such that the patient is unable to sit, stand, or walk without assistance

^b^More than two episodes within 24 h

^c^Including recurrent or prolonged bleeding from the nose, gums, or venipuncture sites; hematemesis or melena

For all 84 children, prostration (66.6%) and multiple convulsions (48.8%) were the most frequent clinical signs of severity. Jaundice (41.6%) and respiratory failure (25%) were significantly more frequent among children who died. Most patients (57.1%) exhibited hyperlactatemia (lactate > 5 µmol/L). More than one-third (38.1%) of the children presented with severe anemia (hemoglobin < 50 g/L, hematocrit < 15%), and one-third had hypoglycemia (glucose < 2.2 mmol/L) at admission.

### Coma etiologies

Table 3 and Additional file 1: Fig. S2 present the etiologies and outcomes of the comas.

**Table 3:** Coma etiologies and outcomes (*n* = 84)

Diagnosis	Cerebral malaria	Co-infection	Non-malarial coma
Number	70	8	6
Etiological details (outcome)		1 *Staphylococcus aureus* bacteremia (survived) 1 *Streptococcus* bacteremia (died) 1 WNV encephalitis (survived) 1 HHV6 encephalitis (survived) 1 Adenovirus encephalitis* (died) 1 Aseptic purulent meningitidis (died) 2 measles (1 died, 1 survived)	1 *E. coli* bacteremia* (died) 1 measles (survived) 1 dengue (survived) 3 undetermined (survived)
Outcome: died	20 (28.6%)	4 (50%)	1 (16,7%)

Data are numbers (%) of children, unless otherwise indicated

*WNV* West Nile virus, *HHV6* human herpesvirus 6

*These patients also had measles specific IgM antibodies

Of the children diagnosed with malaria or co-infection, 16 had parasitemia > 10% (8 of whom died).

Of the five children with measles-specific IgM antibodies, three were unvaccinated (in the entire cohort, 23/84 were not vaccinated against measles).

We could not determine the etiology of the coma in three children, who presented with fever; all received antibiotics, two received anticonvulsants, and one received artesunate. All three improved rapidly; on day 2, their BCS was 5 and they no longer had fevers. They were discharged from the hospital a few days later.

### Treatments before admission

Between disease onset and the day of admission for coma, only 27/78 (34.6%) children with a final diagnosis of malaria or co-infection received anti-malarial drugs. Of these, 6 were treated with artemisinin-based combination therapy (none died), and 21 received other drugs (quinine, *n* = 13; chloroquine, *n* = 5; intramuscular artemether, *n* = 3) of whom 8 died. Of the 51 children who did not receive antimalarial drugs before admission, 16 died.

Children who survived received more antibiotics before admission than did those who died (Table 1).

### Treatments after admission

All but four patients received antimalarial drugs. Of the four, two had non-malarial comas and two died rapidly after admission (one CM, one coinfection with *Streptococcus* bacteremia). Most (58/84) of the children received blood transfusions and 38 (45.2%) received antibiotics at admission; 33 (39.3%) received ceftriaxone. Thirty-one (36.9%) children received anti-epileptic drugs in the hospital.

### Length of hospital stay

Of the 25 patients who died, 20 died within the first 24 h (CM, 16; coinfection, 3; non-malarial coma, 1). The length of stay was consequently shorter for the children who died (mean: 0.67 ± 0.87 days) than for those who survived (mean: 6.97 ± 3.1 days, *P* < 0.001).

### Predictive factors of mortality

Logistic regression analyses were performed to identify factors associated with mortality during hospital care, regardless of the coma etiology. The predictors of death were hyperlactatemia > 5 µmol/L [odds ratio (*OR*) = 8.6; 95% confidence interval (*CI*): 2.03–36.1] and jaundice or its biological equivalent (*OR* = 5.1; 95% *CI*: 1.49–17.30). Antibiotic administration before hospital admission (*OR* = 0.1; 95% *CI*: 0.02–0.85) and vaccination against yellow fever (*OR* = 0.2, 95% *CI*: 0.05–0.79) protected against mortality (Table 4).

**Table 4:** Factors predictive of death in the multivariate analysis

Variable^a^	*OR*	95% *CI*	*P*-value
Hyperlactatemia (lactate > 5 µmol/L)	8.6	2.03–36.1	0.004
Jaundice or increased bilirubin (> 50 µmol/L)	5.1	1.49–17.30	0.009
Antibiotics before admission^b^	0.1	0.02 –0.85	0.03
Yellow fever vaccination	0.2	0.05 – 0.79	0.02

*OR* odds ratio, *CI* confidence interval

^a^Adjusted by center

^b^Between disease onset and admission

## Discussion

We attempted to fill a gap of knowledge on the etiologies, clinical history, clinical and biological profiles, management and outcome of non-traumatic coma in small children from West Africa, a part of the world where access to investigations is limited most of the times to malaria diagnosis. Our prospective study was performed in teaching hospitals and specifically designed to decipher the etiologies of non-traumatic comas, with the help of an independent panel of experts in order to limit the risk of misdiagnosis, provided that non malarial etiologies could represent a substantial part of the cases. We identified only six children with no evidence of malaria. Three of them had a potentially lethal infection [measles, dengue fever, and *E. coli* bloodstream infection (BSI)]. The remaining three had no evidence of meningitis or BSI and had a favorable outcome. These findings contrast with those from other parts of the world where non-infectious (metabolic, vascular) etiologies of comas are more prevalent [15, 16].

A study performed in 2013 in Malawi showed that adenovirus infection is the most common cause of viral CNS infection leading to hospitalization or death. Our results do not corroborate this finding. Five types of viruses, including two arboviruses (one dengue virus and one WNV), were identified in eight children. A measles epidemic occurred during the study period, and we found five children with specific measles IgM as indirect diagnosis of measles, three of whom were coinfected with malaria. The infection could have been prevented in the 3 unvaccinated children*.* The first confirmed cases of dengue fever acquired in Benin were reported in 2010; since then, few cases have been reported [17]. Endemicity of WNV in animals and humans has been reported in Nigeria, a country neighboring Benin [18], but we found no case reports of WNV infections from Benin in the literature, including in returning travelers: so it will be the first report. While dengue is transmitted during the day by the bite of a mosquito of the genus *Aedes*, WNV is transmitted by mosquitoes of the genus *Culex* which have a nocturnal activity like the anopheles which transmit malaria. The use of mosquito nets thus ensures a prevention of the transmission of these 2 infections. In our study, mortality was higher in children with coinfections, but the small number of children precluded drawing firm conclusions. According to a large study on the role of viral CNS coinfections in CM, viral CNS infections are unlikely to contribute to coma [19].

Overall, three BSIs were identified at admission (two coinfections with malaria, one of which was potentially nosocomial). Therefore, tracking BSI is essential in the management of non-traumatic coma. We assume that the child with malaria and an aseptic purulent meningitis coinfection died of meningitis. In a systematic review of invasive bacterial coinfection in African children with *P. falciparum* malaria, the proportion of BSI was estimated at 6.4% [20], and this proportion was not higher in the study setting. Because it is recommended that all children presenting with severe malaria in areas of intermediate and high transmission receive broad-spectrum antibiotics in addition to antimalarial therapy [21], we believe that this also applies to the management of non-traumatic coma, although only a minority of the children was prescribed antibiotics in this study.

CM was the most common cause of non-traumatic coma. In this part of Africa, as well as in other areas endemic for malaria, public health policy efforts have focused on implementing strategies for malaria prevention (e.g., distribution of bed nets) and severe malaria prevention (e.g., affordable, efficient drugs for uncomplicated malaria). Despite this, the remaining substantial burden of severe disease related to malaria should not be neglected. It has been advocated that continuing investment for clinical research on severe malaria is needed [22]. Health policies should also not overlook the need for health facilities that can provide adequate care to children with life-threatening forms of the disease. This is especially true when it comes to access to blood transfusions [23]. Among the planned standardized care procedures and given the large proportion of children with severe anemia in this study, blood transfusions were the most challenging treatment to provide to these children in an emergency. This is an important issue, with many children who had hemoglobin concentrations above the currently recommended transfusion threshold receiving as many transfusions as possible in both hospitals, because this is reportedly associated with improved survival in children with coma and hyperlactatemia [24].

A large majority of the children reported one or more visits with a healthcare professional before admission. For most children in whom CM was eventually diagnosed, missed opportunities for early and effective antimalarial treatment were found. Effort is thus needed to enhance access to affordable and efficient drugs for uncomplicated malaria [8, 25].

Our study also provides information on the risk factors of early death in this population, some of them being unexpected. Mortality remained high despite access to standardized care including intravenous artesunate, and broad-spectrum antibiotics, performed by an experienced teaching hospital staff. Most deaths occurred within a few hours of admission. This is likely because this study involved the most severe forms of CM in the majority of children, sepsis, and other life-threatening diseases. However, malaria and/or another infection were found in all children who died. Some of these children could have benefited from intensive care. As in many other low-income countries [26], there is a growing appreciation of the importance of pediatric acute care at the study sites. This is why we evaluated characteristics at admission associated with mortality.

Among the biological anomalies encountered in sepsis, regardless of its etiology, Jaundice and/or increased bilirubin, as well as lactate above 5 mmol/L at admission were the strongest predictors of subsequent death. This is not surprising because these are additional severity criteria for coma in severe malaria [27]. Furthermore, an increased bilirubin level increases the sequential organ failure assessment score [28], and the total bilirubin and lactate levels are key parameters in a validated mortality risk model for pediatric sepsis [29].

Unexpectedly, a history of antibiotic intake declared before admission and a vaccination against yellow fever were predictors of a better outcome in these children. Antibiotics might have had a protective effect against mortality because some of the children had a bacterial monoinfection or coinfection with malaria. Most deaths occurred shortly after admission, before any emergency antibiotic prescription could have been effective. Antibiotics given before admission may play a role in protecting against a fatal outcome, including in children with CM. It may also be an indicator of a better overall management of these children, independently of the treatment administered.

Yellow fever was not among the diseases screened for in this study; therefore, it is not possible to state that no yellow fever co-infection existed. More frequent yellow fever co-infections in the children who died might have worsened their prognosis. Another explanation for the protective effect of the yellow fever vaccine could be a non-specific protective effect of this live vaccine. Non-specific effects of live vaccines have been described for the smallpox, BCG, oral polio, and measles vaccines in experimental and observational studies [30]. In Burkina Faso, before mass vaccinations against meningitis and measles, a measles and yellow fever vaccine was associated with reduced mortality [31]. In Guinea-Bissau, measles and yellow fever vaccines were associated with stronger beneficial effects in girls than in boys [32]. In South Benin, the measles and yellow fever vaccines are administered in principle on the same day, but shortages sometimes occur, which may explain why the respective vaccination rates sometimes differ between the two vaccines, leading to a discrepancy in their protective effect against mortality in our analysis.

This study has several limitations. Data on clinical history, based on parents/guardians interview at admission and health booklet review may have not been exhaustive. We raised no brain imaging data and we may have missed for example cerebral tumors or bleeding as a cause of coma. The study was not designed to diagnose measles: there was no repeated serologies, and the diagnosis is less robust than for other etiologies. However, we are confident that the diagnoses of CM and infections by non-malaria pathogens have been quite exhaustive and reliable, due to the high quality of laboratory testing and the use of a panel of experts to ensure the best classification of the patients. Finally, despite the fact that this study ranks among the largest cohorts of severe comas in African children and the only one performed in West Africa, it may have lacked power to extensively identify the factors associated with death outcomes. Although NeuroCM was mainly a case–control study to explore inflammation in comatose vs non-comatose malaria infected children [11], not specifically designed to define the risk factors of early death in the former population, it still allowed to clearly evidence significant variables, usual (hyperlactatemia and elevated bilirubin) as well as more novel (antibiotics intake, yellow fever immunization).

## Conclusions

Cerebral malaria was the most common cause of non-traumatic coma in the study area and was associated with a high mortality rate. For most children, missed opportunities to receive early and effective antimalarial treatment were detected. A history of antibiotic intake declared before admission and vaccination against yellow fever were protective against death in this population. Efforts against malaria should not overlook the need for health facilities that can provide adequate care to children with life-threatening forms of the disease.

## Supplementary Information

**Additional file 1: Figure S1.** Map of Benin with focus on Cotonou and the 2 study sites. **Figure S2.** Coma etiologies: graphical presentation.

## Data Availability

The data presented in this study are available on reasonable request from the corresponding author.

## Abbreviations

BCG: Bacille Calmette-Guérin
BCS: Blantyre coma score
BSI: Bloodstream infection
*CI*: Confidence interval
CM: Cerebral malaria
CNS: Central nervous system
DTP: Diphtheria, tetanus, and pertussis vaccine
HepB: Hepatitis B vaccine
HHV6: Human herpesvirus 6
Hib: Hemophilus influenzae type b vaccine
HIV: Human immunodeficiency virus
MMR: Measles, mumps, and rubella vaccine
OPV: Oral polio vaccine
*OR*: Odds ratio
PCR: Polymerase chain reaction
PCV13: Pneumococcal conjugate vaccine (13-valent)
RDT: Rapid diagnostic test
*SD*: Standard deviation
SM: Severe malaria
WNV: West Nile virus
YF: Yellow fever vaccine
