# Supplementary material for: Non-traumatic coma in young children in Benin: are viral and bacterial infections gaining ground on cerebral malaria?
Source: Infect Dis Poverty. 2022 Mar 14;11:29. doi: 10.1186/s40249-022-00956-2 (PMC8919613; doi:10.1186/s40249-022-00956-2)
Supplement: Supplementary file 1 — Additional file 1: Figure S1. Map of Benin with focus on Cotonou and the 2 study sites. Figure S2. Coma etiologies: graphical presentation. [file 40249_2022_956_MOESM1_ESM.docx]

**Additional materials – Figures legends**

**
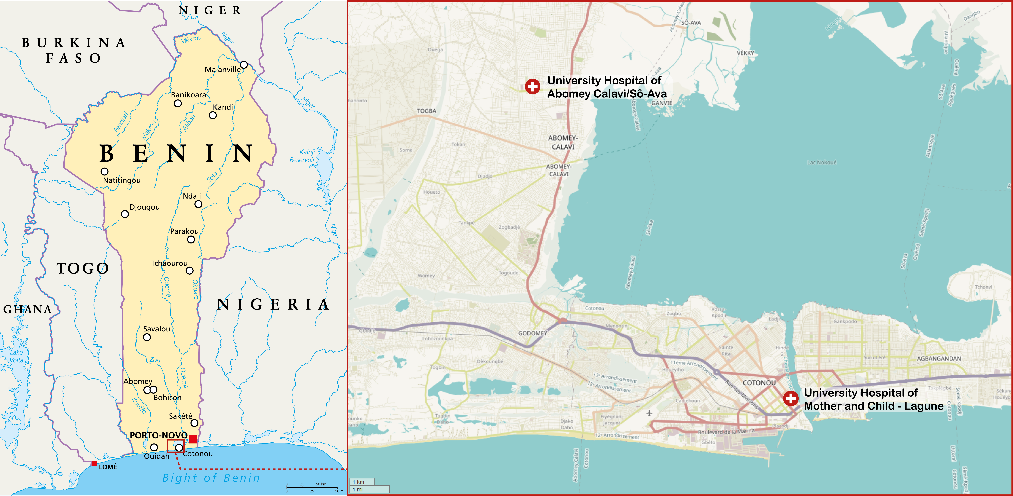
**

**Figures S1. Map of Benin with focus on Cotonou and the 2 study sites.**

Cotonou/Abomey-Calavi map: OpenStreetMap®


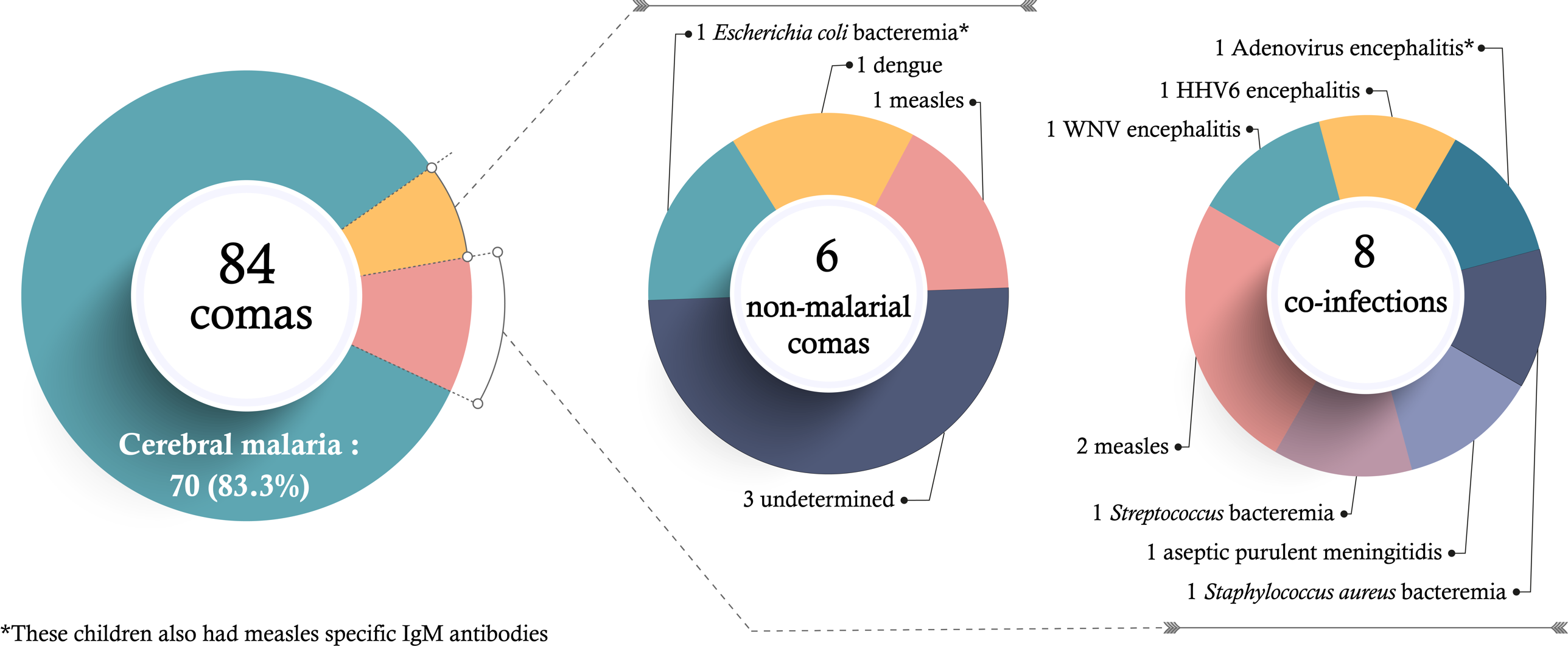


**Figure S2. Coma etiologies - graphical presentation**

Abbreviations: WNV, West Nile virus; HHV6, Human herpesvirus 6
